# The role of DEAD- and DExH-box RNA helicases in neurodevelopmental disorders

**DOI:** 10.3389/fnmol.2024.1414949

**Published:** 2024-08-01

**Authors:** Johannes Lederbauer, Sarada Das, Amelie Piton, Davor Lessel, Hans-Jürgen Kreienkamp

**Affiliations:** ^1^Institute of Human Genetics, University Hospital Salzburg, Paracelsus Medical University, Salzburg, Austria; ^2^Institute of Human Genetics, University Medical Center Hamburg-Eppendorf, Hamburg, Germany; ^3^Department of Translational Medicine and Neurogenetics, Institute of Genetics and Molecular and Cellular Biology, Strasbourg University, CNRS UMR7104, INSERM U1258, Illkirch, France

**Keywords:** stress granules, P-bodies, miRNA, translation, R-loop

## Abstract

Neurodevelopmental disorders (NDDs) represent a large group of disorders with an onset in the neonatal or early childhood period; NDDs include intellectual disability (ID), autism spectrum disorders (ASD), attention deficit hyperactivity disorders (ADHD), seizures, various motor disabilities and abnormal muscle tone. Among the many underlying Mendelian genetic causes for these conditions, genes coding for proteins involved in all aspects of the gene expression pathway, ranging from transcription, splicing, translation to the eventual RNA decay, feature rather prominently. Here we focus on two large families of RNA helicases (DEAD- and DExH-box helicases). Genetic variants in the coding genes for several helicases have recently been shown to be associated with NDD. We address genetic constraints for helicases, types of pathological variants which have been discovered and discuss the biological pathways in which the affected helicase proteins are involved.

## Introduction

RNA helicases use energy from the hydrolysis of ATP to unwind double stranded sections of RNA/RNA or RNA/DNA hybrids, and may also assist in the restructuring of RNA/protein complexes (ribonucleoproteins, RNPs). They carry out essential cellular functions, many of which are conserved from yeast to humans (Linder and Jankowsky, 2011). Accordingly, the distinct families of these helicases are also highly conserved throughout eukaryotic evolution (Pyle, 2008). Six major helicase superfamilies have been identified (SF1-SF6) which have distinct functions in several aspects of DNA and RNA metabolism (Fairman-Williams et al., 2010). While members of SF3-6 superfamilies are active in a “toroidal,” hexameric form, SF1 and SF2 are active as monomers. SF1 and SF2 helicases are rather similar to each other, but individual differences in conserved helicase core motifs allow for a clear differentiation between the two families (Gorbalenya and Koonin, 1993; Tanner and Linder, 2001; Fairman-Williams et al., 2010). We will focus here on the DEAD-and DExH-box helicase families which are part of the SF2 superfamily, and which in humans consist of 54 different members. While it appears surprising that our transcriptome (and genome) requires such a diversity of different helicase activities, it should be noted that every single aspect, e.g., of the life cycle of an RNA requires in most cases not only one, but several different helicases. Quite often a complete knockout of one of the RNA helicase coding genes is lethal early in mouse development (Li et al., 2014; Zheng et al., 2015; Kim et al., 2022). On the other hand, heterozygous, loss-of-function or missense variants in helicase genes are often associated with a neuronal, or neurodevelopmental phenotype in humans (Snijders Blok et al., 2015; Lessel et al., 2017; Balak et al., 2019). This may reflect the specific requirements of the developing nervous system for precise regulation of gene expression and RNA metabolism.

## Neurodevelopmental disorders (NDDs)

NDDs represent a large, clinically and genetically, heterogeneous group of human disorders with an onset in the neonatal or early childhood period. NDDs include intellectual disability (ID), autism spectrum disorders (ASD), attention deficit hyperactivity disorders (ADHD), seizures, various motor disabilities and abnormal muscle tone (Morris-Rosendahl and Crocq, 2020). NDDs are usually characterized by impairments in cognition, communication, adaptive behavior and psychomotor skills.

NDDs are often associated with Mendelian, single genetic events such as chromosomal rearrangements, copy number variations, small insertions/deletions, nonsense or missense variants. NDDs have been estimated to affect 3% of the general population (Gilissen et al., 2014), with 0.5% of all newborn affected by severe ID (Parenti et al., 2020). Although each of the underlying genetic causes of NDD is rare, their accumulated number is high enough worldwide to cause a serious socio-economic problem for health care systems. The genetic testing now routinely relies on next generation sequencing (NGS) techniques, i.e., whole exome/whole genome sequencing, or a panel based approach focused on known NDD genes. Identification of novel Mendelian, genetic causes requires a complex process, which includes evaluation of databases of human genetic variations such as the gnomAD database (Chen S. et al., 2024), identification of similarly affected individuals harboring similar genetic variants and in most instances, confirmatory functional analyses. Due to the increased use of NGS, the last decade has seen a tremendous increase in identification of novel genetic causes for NDDs. However, for the majority of NDDs two main challenges remain: reliable assessment of the pathogenicity of identified variants and meaningful clinical interventions. Thus, there is currently an urgent need for improved understanding of NDD pathology (Gilissen et al., 2014; Niemi et al., 2018; Parenti et al., 2020).

## Which genes are affected in NDD patients?

One might have thought that genes involved in neuron-specific functions would contribute to the prevalence of NDDs. Indeed, pathogenic variants in genes coding for synaptic proteins have been implicated in autism spectrum disorders and in ID (Bourgeron, 2009). However, a more quantitative analysis points to genes coding for proteins involved in control of the different steps of the gene expression pathway, such as transcriptional regulators, splicing factors, translational regulators or aspects of miRNA pathways (Greene et al., 2023). In fact, the most prevalent cause for ID now appears to be a pathogenic variant affecting the non-coding RNA RNU4-2, which is a component of the spliceosome (Chen Y. et al., 2024; Greene et al., 2024). It should be noted that in contrast to other tissues, the nervous system consists of an amazingly large number of different cell types. These can be differentiated based on single cell transcriptomics. Thus, one recent study identified 461 clusters of different cell types in the human brain, with 3,313 individual subclusters of cell types defined by a specific pattern of transcripts (Siletti et al., 2023). Due to this extreme transcriptomic diversity, the developing brain may require the precise regulation of gene expression pathways much more than other tissues. Furthermore, neurons engage in localized protein synthesis both in dendrites and in axons (Steward and Schuman, 2001; Cajigas et al., 2012). Local protein synthesis in dendrites, close to postsynaptic sites, is believed to contribute to synaptic plasticity, i.e., to activity dependent changes in synaptic strength which should be specific to those synapses which have been activated (Kindler and Kreienkamp, 2012; Sun et al., 2021). Local protein synthesis in axons is necessary due to the long distance of axon terminals from the cell body, making somatic protein synthesis followed by protein transport to synaptic terminals too slow for replenishment of and adaptation of protein levels. Many mRNAs have been shown to be not only present, but also translated locally near pre-or postsynaptic sites (Hafner et al., 2019; Glock et al., 2021). This concept entails transport of ribosomes, tRNAs and mRNAs to dendrites or axons, localized control of translation as well as the eventual degradation of the localized mRNAs. Indeed, specific structures involved in the regulation of mRNA translation such as stress granule components and P-bodies have been detected in neuronal axons and dendrites (Shiina et al., 2005; Cougot et al., 2008; Zeitelhofer et al., 2008; Sahoo et al., 2018; Lessel et al., 2020). Again, the requirements for proteins involved in RNA metabolism appear to be more complex in neurons when compared to non-neuronal cells. RNA helicases of the DExH/DEAD-box families constitute a large group of proteins which may be present in dendrites (Kanai et al., 2004), which contribute to these processes, and which may cause neurological problems upon the occurrence of damaging alterations in their coding genes.

## Structure of DExH/DEAD-box helicases

**Figure 1:**
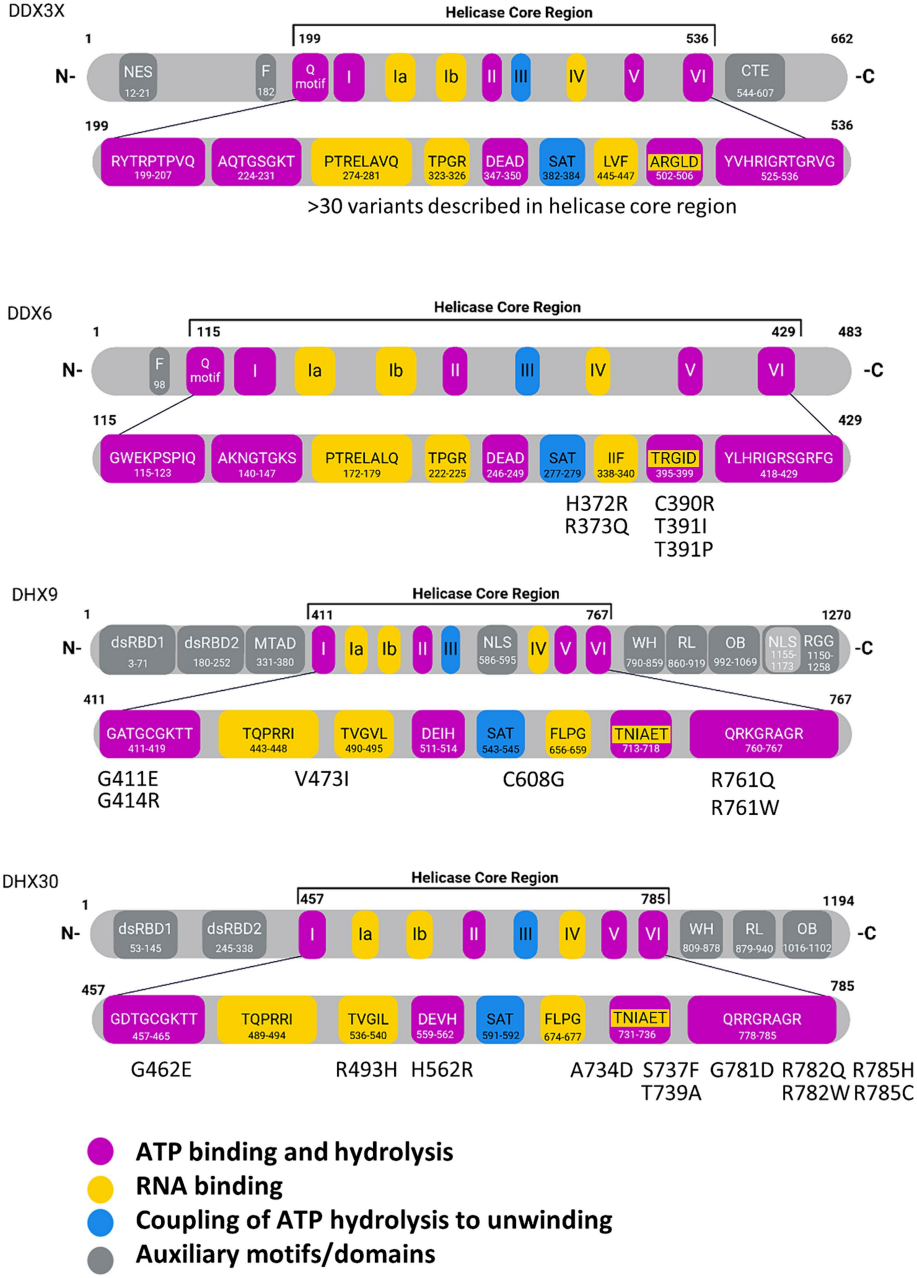
Domain structures of individual RNA helicases involved in NDDs. In the enlargement of the helicase core regions, individual core motifs are indicated with amino acid sequences and positions, based on Uniprot entries for the human proteins. Missense variants identified in NDD patients are indicated for DDX6, DHX9 and DHX30. For DDX3X, more than 30 variants have been described in the helicase core region. Motif V has been implicated in RNA and ATP binding. WH, Winged-helix; RL, Rachet-like; OB, Oligosaccharide binding; Znf, Zinc finger; dsRBD, double stranded RNA binding domain; mtad, minimal transactivation domain; NLS, nuclear localization signal; NES, nuclear export signal; NTD, N-terminal domain; RGG, arginine-glycine–glycine domain. Created with Biorender.com.

The core functions of DexH/DEAD-box RNA helicases (ATP binding and hydrolysis, nucleic acid binding and unwinding) are carried out by two adjacent core domains which show structural similarity to the recombination protein RecA. Within these helicase core domains, up to 14 conserved helicase core motifs (HCMs) can be identified (Fairman-Williams et al., 2010). Out of these, Ia – Ib, IV, IVa, V and Vb bind nucleic acid substrates. HCMs Q, I, II, IIIa and VI are involved in ATP binding and hydrolysis. Interestingly, not all HCMs are present in all members of these helicase families (Fairman-Williams et al., 2010). Thus, the Q-HCM, a 9 amino acid (aa) sequence containing an invariant glutamine residue along with a conserved phenylalanine residue 17 aa further upstream, is found only in DEAD-box RNA helicases. The namesake DEAD or DExH sequence motifs constitute motif II. In motif II, specifically the Asp-Glu part is involved in coordinating the ATP associated Mg^2+^ ion, and in positioning the water molecule which performs the ATP hydrolysis. In 3D structures of both DEAD-box as well as DExH box proteins, the side chains of the C-terminal Asp/His residues of motif III are in direct contact to the Ser and Thr side chains of motif III (sequence SAT), which couples NTP binding and hydrolysis to nucleic acid binding and unwinding (Tanner et al., 2003; Cordin et al., 2006; Figure 1; see Supplementary Figure S1 for a complete view on human DEAD box proteins).

DExH helicases are processive helicases which move along a dsRNA substrate, capable of performing several unwinding steps along the way. In contrast, DEAD-box proteins dissociate from their RNA substrates after a single unwinding step (Bohnsack et al., 2023). The unique C-terminal domains of DExH helicases, i.e., the winged-helix (WH) and ratchet-like domains (often described together as helicase associated 2 or HA2 domain), as well as the oligonucleotide/oligosaccharide-binding (OB)-fold domain are relevant here (Murakami et al., 2017; Jagtap et al., 2023; Figure 1; see Supplementary Figure S2 for a complete view on human DExH box proteins). For these helicases, HA2 and OB motifs are integral parts of the helicase function as they contribute to a tunnel for single stranded RNA. In the case of the *Drosophila maleless* (MLE) helicase, a member of the DExH family which has been studied in much detail, the core RecA domains bind the RNA substrate via non sequence specific interaction with the sugar/phosphate backbone. In contrast, the OB domain is involved in base-specific contacts at the 5′ end of the tunnel. Here, also the second of the two N-terminal dsRBD domains (dsRBD2) is essential for activity as it is involved in regulating helicase activity, and also provides for proper positioning of dsRNA substrates at the entrance of the RNA tunnel (Prabu et al., 2015; Jagtap et al., 2023).

Other accessory domains, including additional dsRNA binding domains or RNA recognition motifs (RRMs), are typically not conserved within a given family of these RNA helicases. Both, N-and C-terminal domains frequently determine the integration of helicase proteins into larger functional complexes and are therefore highly relevant for the physiological function.

## Multiple functions of RNA helicases during the RNA life cycle

**Figure 2:**
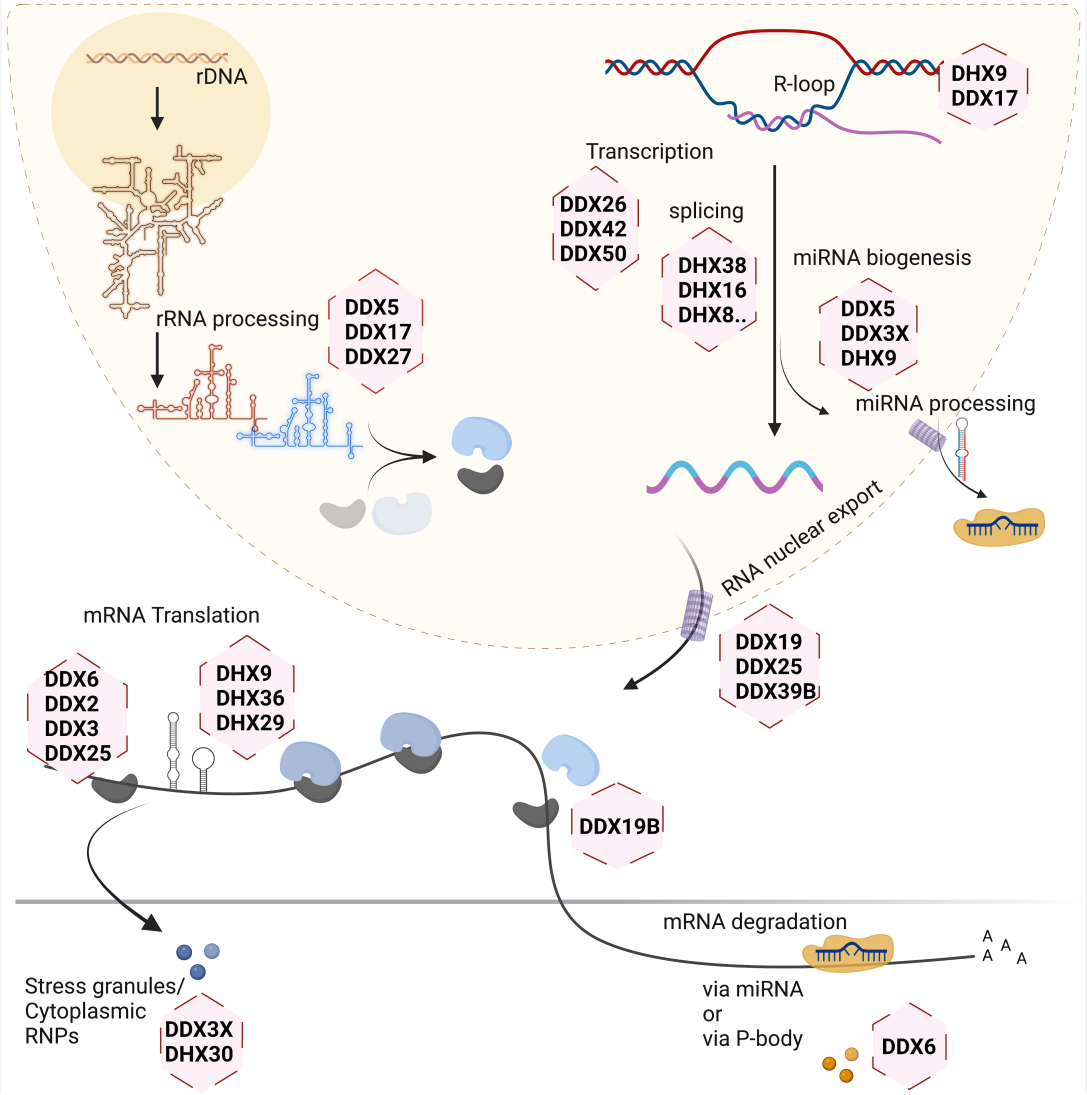
Involvement of RNA helicases in different stages of eukaryotic RNA metabolism. Created with Biorender.com.

Different stages of the RNA life cycle are shown in Figure 2. Transcription produces the crude RNA that will go through several interactions with various proteins or different kinds of RNA in its path that shapes its journey. Transcription is coordinated by several RNA helicases which act as coactivators or corepressors by binding to key transcriptional machinery (Rajendran et al., 2003; Rossow and Janknecht, 2003; Yan et al., 2003). This is relevant for both Pol1 and Pol2-transcribed genes, as has been shown for DDX21 which associates with genes coding for ribosomal RNA, as well as ribosomal proteins and positively regulates their transcription (Calo et al., 2015). In addition, several RNA helicases (e.g., DDX1, DDX17 and DHX9) have been suggested to contribute to the formation and resolving of so-called R-loops. These three-stranded nucleic acids, consisting of an RNA–DNA hybrid and a displaced single-stranded DNA, occur during transcription and DNA replication, and must be resolved to avoid epigenetic misregulation (Al-Hadid and Yang, 2016) and genome instability (Yang et al., 2023).

Ribosome biogenesis starts in the nucleolus, with a few final steps occurring in the cytoplasm. As it involves the dynamic rearrangement of ribonucleoprotein complexes, it requires a multitude of RNA helicases to help avoid undesirable configuration of RNA and its interactions. In the yeast model system, at least 19 RNA helicases are required for the maturation of ribosomes, and even more helicases are involved in this process in human cells (Martin et al., 2013). A single large precursor rRNA molecule undergoes systematic cleavage by endo-and exonucleases along with several other hundreds of transacting factors to produce the mature rRNAs. The main roles of the DExH/DEAD-box helicases during this process are to mechanistically fold the precursor rRNA for formation of several RNP complexes, careful removal of specific small nuclear RNAs guiding rRNA folding, and mediating structural alteration in ribosomal subunits during the ribosome assembly (Jalal et al., 2007; Martin et al., 2014; Kellner et al., 2015; Xing et al., 2019).

Splicing of newly transcribed pre-mRNAs to form the mature mRNA involves structural rearrangements and folding which requires assistance by several RNA helicases. Studies of yeast as well as human spliceosomes have shown that at least eight RNA helicases act in a sequential manner, with seven of them belonging to DExH/DEAD-box families (Fleckner et al., 1997; Gencheva et al., 2010; English et al., 2012; Zanini et al., 2017; Hug et al., 2022; Obuća et al., 2022). Often these helicases share more than one role in the cellular system. For example, RNA helicase DDX39B is part of the spliceosome affecting splicing and also regulates nuclear export of mRNAs by being part of the TREX mRNA export complex (Li et al., 2005; Kota et al., 2008; Hautbergue et al., 2009; Shen, 2009). On the other hand, DDX39B also contributes to translation by regulating pre-ribosomal RNA levels (Awasthi et al., 2018).

The translation machinery associates with most of the major kinds of RNA in the cell. This is one process that involves the three major types of RNA in the cell: the mRNA, rRNA, and tRNA. So naturally, the helicases involved here inter-share their roles to maintain a coherent system. The eIF4A RNA helicase (also known as DDX2) constitutes one of the smallest DEAD-box helicases. Its evolutionary conservation is due to its indispensable role for translation initiation, to unwind the 5’UTR of mRNA, thus facilitating scanning of the 5’UTR by small ribosomal subunits to identify the start codon (Pause and Sonenberg, 1992). Other RNA helicases such as DDX3, DHX9 and DHX36 are needed to overcome highly structured 5’UTRs on large transcripts during the scanning process (Sheng et al., 2006; Lee et al., 2008; Calviello et al., 2021). Another RNA helicase recently found crucial for protein biosynthesis is DHX19, which assists in formation of the termination complex and release of the newly formed protein from the ribosomal complex (Mikhailova et al., 2017).

Several regulatory mechanisms act on mRNAs to control translation. Translational shutdown upon various cellular stresses leads to sequestration of mRNAs in large protein/RNA complexes within the cytoplasm called stress granules (SGs). Several RNA helicases such as DDX3X, DHX30, DHX36 act in the assembly, and possibly also in the disassembly or clearance of SGs (Chalupnikova et al., 2008; Valentin-Vega et al., 2016; Sauer et al., 2019; Mannucci et al., 2021). In addition, mRNAs may be degraded in processing (P-) bodies, which also exist under basal (non-stressed) conditions and are associated with miRNA dependent silencing of mRNAs. The helicase DDX6 plays major role in P-bodies and is involved in several aspects of mRNA degradation (Nissan et al., 2010; Ostareck et al., 2014; Rouya et al., 2014; Weber et al., 2024).

Both SGs and P-bodies, together with nucleoli and nuclear speckles, constitute membrane-less organelles which perform a large portion of cellular RNA processing. These cellular bodies are held together by a multitude of interactions of RNAs with RNA binding proteins, and frequently involve a biomolecular condensation process termed liquid-/liquid phase separation (LLPS) (Dorner and Hondele, 2024). Proteins can contribute to condensate formation through their various interaction domains, but also through larger segments of intrinsically disordered regions (Rosa et al., 2024). Most if not all DEAD-and DExH helicases are present in such condensates at some point of the RNA life cycle, and they are probably needed for both the assembly as well as the disassembly of such organelles (Hondele et al., 2019). This is exemplified by the two sexually dimorphic DDX3 variants, DDX3x and DDX3y. Here, it was recently shown that the disordered region in DDX3y promoted LLPS more strongly, while its weaker ATPase activity was less active in SG dsassembly. This leads to increased LLPS, reduced translation and increased mRNA aggregation, e.g., under stress in a sex-specific manner (Shen et al., 2022). Aberrant formation of stress granules is an important feature of NDD associated pathogenic variants in DHX30 and DDX3x (Lessel et al., 2017; Lennox et al., 2020; Mannucci et al., 2021).

### Involvement of DExH/DEAD-box RNA helicases in NDDs

In order to provide a detailed overview of the DExH/DEAD-box RNA helicases involved in NDDs we first queried the Online Mendelian Inheritance in Man (OMIM)^1^ database. OMIM is a comprehensive compendium that continuously updates human genes and their association to human genetic disorders according to the Human Genome Organization (HUGO) Gene Nomenclature Committee (HGNC).^2^ This search revealed nine RNA helicase genes, that can be regarded as definitely associated with a human disorders. Out of these six genes were associated with a primary neurodevelopmental disorder. In more detail, missense and loss-of-function variants in *EIF4A2* (*DDX2B*) are associated with the Neurodevelopmental disorder with hypotonia and speech delay, with or without seizures (NEDHSS, #620455) (Paul et al., 2023). Similarly, a broad spectrum of variants in *DDX3X* is associated with Syndromic X-linked intellectual developmental disorder of the Snijders Blok type [MRXSSB, #300958 (Snijders Blok et al., 2015)]. Missense variants in *DDX6* and *DHX16* are associated with Intellectual developmental disorder with impaired language and dysmorphic facies [IDDILF, #618653 (Balak et al., 2019)] and Neuromuscular oculoauditory syndrome [NMOAS, #618733 (Paine et al., 2019)], respectively. Missense variants in *DHX30* are associated with Neurodevelopmental disorder with variable motor and language impairment (NEDMIAL, #617804), with loss-of-function variants causing a milder phenotype (Lessel et al., 2017; Mannucci et al., 2021). Missense variants in *DHX37* are associated with Neurodevelopmental disorder with brain anomalies and with or without vertebral or cardiac anomalies [NEDBAVC, #618731 (Paine et al., 2019)]. In addition, *DDX11*, *EIF4A3* and *DDX59* have been associated with complex human disorders that involve a variable degree of neurodevelopmental delay. These genes have bene associated with Warsaw breakage syndrome [#601150 (van der Lelij et al., 2010)], Robin sequence with cleft mandible and limb anomalies [#268305 (Favaro et al., 2014)] and Orofaciodigital syndrome V [OFD5, #174300 (Shamseldin et al., 2013)], respectively (Table 1).

**Table 1:** DExH/DEAD-box RNA helicases associated with NDDs.

Gene name	Inheritance	OMIM	Evidence (SysNDD)
*DDX1*	AR	No	Single affected individual
*EIF4A2 (DDX2B)*	AD, AR?	Neurodevelopmental disorder with hypotonia and speech delay, with or without seizures (NEDHSS, #620455)	*De novo* variants, 1 individual with a homozygous variant
*DDX3X*	XLD, XLR	Syndromic X-linked intellectual developmental disorder of the Snijders Blok type (MRXSSB, #300958)	*De novo* variants in females, inherited missense variants in affected males
*DDX6*	AD	Intellectual developmental disorder with impaired language and dysmorphic facies (IDDILF, #618653)	*De novo* missense variants
*DDX11*	AR	Warsaw breakage syndrome (#601150)	Homozygous and compound heterozygous variants
*DDX17*	AD?	No	*De novo* truncating variants in 2 individuals
*DDX23*	AD?	No	*De novo* missense variants
*DDX24*	AD?	No	Balanced translocation in a single individual
*DDX47*	AD?	No	One individual with comp het missense variants
*EIF4A3 (DDX48)*	AR	Robin sequence with cleft mandible and limb anomalies (#268305)	Homozygous and compound heterozygous variants
*DDX50*	AD?	No	Inframe deletion in a single individual
*DDX54*	AR?	No	Two individuals with hom or comp het missense variants
*DDX59*	AR	Orofaciodigital syndrome V (OFD5, #174300)	Homozygous and compound heterozygous variants
*DHX9*	AD?	No	*De novo* variants
*DHX16*	AD	Neuromuscular oculoauditory syndrome (NMOAS, #618733)	*De novo* variants
*DHX30*	AD, AR?	Neurodevelopmental disorder with variable motor and language impairment (NEDMIAL, #617804)	*De novo* variants, single individual with homozygous variant
*DHX34*	AD?, AR?	No	Single individual with a *de novo* variant, two individuals with homozygous variants
*DHX37*	AR, AD?	Neurodevelopmental disorder with brain anomalies and with or without vertebral or cardiac anomalies (NEDBAVC, #618731)	Five individuals with AR, two individuals with *de novo* variants
*DHX58*	AR?	No	One individual with homozygous missense variant

Definitely established genes are marked in black, candidate genes are marked in purple. AD, autosomal dominant; AR, autosomal recessive; XLD, X-linked dominant, XLR, X-linked recessive; ?, not established. OMIM, Online Mendelian Inheritance in Man (www.omim.org) database. SysNDD database (sysndd.dbmr.unibe.ch), last accessed July 2024.

To search for further candidate genes, we next enquired the SysNDD database (sysndd.dbmr.unibe.ch) that curates gene disease relationships in NDDs. This search revealed 10 potential candidate NDD genes, variants in which have been identified in at least one affected individual (Table 1). Out of these, an association of *DHX9* (Calame et al., 2023; Yamada et al., 2023) and *DDX23* (Burns et al., 2021) with a NDD has been published only recently.

The gross majority of genes involved in human NDDs are highly intolerant to genetic variation, both to missense and loss-of-function variants (Samocha et al., 2014). This is nicely exemplified by the *DHX30* gene, being one of the most variation-intolerant genes in the human genome (Lessel et al., 2017). We have therefore utilized the large sequencing data from the Genome Aggregation Database (gnomAD V2.1)^3^ (Karczewski et al., 2020) to document constraint metrics of the 54 human DExH/DEAD-box RNA helicases (Table 2). We were interested in the tolerance to missense variants (missense Z-scores), where a score of >3 is regarded as intolerant, and tolerance to loss-of-function variants [probability of being loss-of-function intolerant (pLI)], where a score of >0.90 is regarded as intolerant (Samocha et al., 2014; Lek et al., 2016). Genes with either high Z score or pLI values, or both, are regarded as strong candidates for disorders caused by heterozygous*, de novo* variants (Samocha et al., 2014). Indeed, out of the already established NDD-related DExH/DEAD-box RNA helicase genes, primarily caused by *de novo* variants, all bear high scores (*DDX3X*, *DDX6*, *DHX16* and *DHX30*). Out of the candidate genes listed in Table 1, *DDX17*, *DDX23* and *DHX9* show high missense Z scores and pLI’s.

**Table 2:** Constraint metrics of DExH/DEAD-box RNA helicases according to gnomAD V2.1.

Gene	Z score	pLI	Gene	Z score	pLI
*DDX1*	1.82	0.99	*DDX47*	0.39	0
*DDX2A*	3.93	1	*DDX48*	4.02	1
*DDX2B*	3.89	1	*DDX49*	0.96	0
*DDX3Y*	2.1	0.96	*DDX50*	2.07	0
*DDX3X*	4.33	1	*DDX51*	−1.41	0
*DDX4*	2.05	1	*DDX52*	0.18	0
*DDX5*	2.76	1	*DDX53*	0.01	0.66
*DDX6*	3.78	1	*DDX54*	0.68	0
*DDX10*	0.3	0	*DDX55*	0.65	0
*DDX11*	−0.22	0	*DDX56*	−0.23	0
*DDX17*	3.87	1	*DDX58*	0.82	0
*DDX18*	0.47	0.14	*DDX59*	0.78	0
*DDX19A*	2.37	0.84	*DHX8*	5.03	0
*DDX19B*	2.22	0	*DHX9*	5.84	1
*DDX20*	0.08	0	*DHX15*	5.63	1
*DDX21*	2.45	1	*DHX16*	3.08	0
*DDX23*	4.62	0.54	*DHX29*	1.93	0
*DDX24*	0.09	0.68	*DHX30*	5.3	1
*DDX25*	1.21	0	*DHX33*	0.46	0
*DDX27*	1.73	0.58	*DHX34*	−0.08	0
*DDX28*	−0.75	0	*DHX35*	0.48	0
*DDX31*	0.03	0	*DHX36*	1.84	0.47
*DDX39*	3.55	1	*DHX37*	1.83	0.99
*DDX41*	2.28	0	*DHX38*	2.67	0
*DDX42*	3.29	1	*DHX40*	3.02	0.76
*DDX43*	1.27	0	*DHX57*	−0.96	0
*DDX46*	5.48	1	*DHX58*	0.52	0

Indicated in yellow are genes already associated with a NDD, in red are the high missense Z scores (>3) and pLIs (Probability of being loss-of-function intolerant; >0.9) indicated.

Neurodevelopmental disorders arise due to various perturbations during brain development (Khodosevich and Sellgren, 2023). Thus, we were also interested in the expression profiles of the DExH/DEAD-box RNA helicases in various brain regions during brain development. For this, we utilized the Human Brain Transcriptome (HBT) database,^4^ which provides transcriptome data for the developing and adult human brain (Kang et al., 2011) in six brain regions (neocortex, hippocampus, amygdala, striatum, mediodorsal nucleus of the thalamus and cerebellar cortex). We documented the mean expression levels at four different time points during development, namely during the embryonic period (TP1; 4 PCW ≤ Age < 8 PCW), late fetal period (TP7, 24 PCW ≤ Age < 38 PCW), neonatal and early infancy period (TP8, 0 M (birth) ≤ Age < 6 M) and early childhood (TP10, 1 Y ≤ Age < 6 Y) (Table 3). Out of the already established NDD-associated DExH/DEAD-box RNA helicase genes, *EIF4A2* (DDX2B), *DDX3X*, *DDX6* and *DHX30*, showed a strong expression during all developmental periods. Genes associated with complex human disorders, *DDX11* and *EIF4A3,* displayed strong expression only at certain periods. *DDX59*, *DHX16* and *DHX37* are not highly expressed during brain development. Out of the candidate genes, *DDX1*, *DDX17*, *DDX24*, *DDX47* and *DHX9* display a strong expression during all developmental periods, whereas DDX23 displays a somewhat lower expression only at TP7.

**Table 3:** Gene expression data of DExH/DEAD-box RNA helicases in 6 brain areas during human development.

	TP 1	TP 7	TP 8	TP 10		TP 1	TP 7	TP 8	TP 10		TP 1	TP 7	TP 8	TP 10
DDX1	10.5	8.5	9	9	DDX28	5.5	4.8	4.8	5	DHX8	8.5	7	7	7
DDX2A	12	11	10	10	DDX31	5.9	5.7	5.3	5.3	DHX9	10.8	8	8	8
DDX2B	10.3	11	11	12	DDX39	7.5	6	5.8	5.8	DHX15	10.5	8.3	8.3	8.3
DDX3X	9.5	8.3	8.5	8.5	DDX41	-	-	-	-	DHX16	7	5.8	5.8	5.8
DDX3Y	8.2	8	7.8	7.8	DDX42	8.5	7.8	7.8	8	DHX29	9	8	9	10
DDX4	3.7	4	4	4	DDX43	3.9	4.1	4.1	4.1	DHX30	9.5	8	9	9
DDX5	11.5	10.2	10.2	10.2	DDX46	9	7	7	7	DHX32	8.7	6.5	6.5	6.5
DDX6	11.8	11.5	11	11	DDX47	9	8	8	8	DHX33	7.5	6.3	6.3	6.3
DDX10	9.2	8	8	8.5	DDX48	10	7.5	8	7.5	DHX34	6	5.8	5.6	5.5
DDX11	7	11	11	10	DDX49	7.2	7	6.9	6.5	DHX35	8	5.7	5.8	5.8
DDX17	12	11	11	10.8	DDX50	8.3	7	7	7	DHX36	9	8	8	8
DDX18	9	6.8	7	6.5	DDX51	6	6	6	6	DHX37	6.3	6	6	6
DDX19A	-	-	-	-	DDX52	10	7.2	7	7	DHX38	7	6.6	6.6	6.6
DDX19B	8.2	7.5	7	7	DDX53	3.7	3.7	3.7	3.7	DHX40	9	6.5	6.3	6.3
DDX20	7.5	5.5	5.8	5.5	DDX54	7.8	6.8	6.8	6.8	DHX57	8.5	6.8	6.3	6.3
DDX21	8.8	6.9	6.8	6.8	DDX55	6.2	6	5.7	5.7	DHX58	4.5	5	5.3	5.5
DDX23	9.8	7.1	8	8	DDX56	8.7	7	7.4	7.3					
DDX24	10	9.5	10	10.4	DDX58	5.7	5.7	5.7	5.7					
DDX25	6.3	7	7.5	7.7	DDX59	6.5	6.1	5.8	5.8					
DDX27	6.5	6.3	6.3	6										

Expression levels of all DExH/DEAD-box RNA-helicases during human development was taken from Human Brain Transcriptome (HBT) database. TP1 – embryonic; TP7 – Late fetal; TP8 – Neonatal and early infancy; TP10 – early childhood; indicated in pink are values ≥8.

Taken together the constraint metrics and brain expression data provide evidence for heterozygous, *de novo* variants in *DDX17*, *DDX23* and *DHX9* as being associated with NDDs. Based on both datasets we suggest that heterozygous, *de novo* variants in *DDX2A*, *DDX42*, *DDX46*, *DHX8*, *DHX15* and *DHX40* might represent additional candidates for this group of disorders. Finally, based on brain expression levels, *DDX1*, *DDX5*, *DDX24*, *DDX47* and *DHX36* might constitute NDD associated genes following an autosomal recessive mode of inheritance. Clearly, further high-throughput sequencing studies are needed to confirm these hypotheses.

Below we provide a brief overview of the four well-studied NDDs associated with pathogenic variants in DExH/DEAD-box RNA helicases.

### DHX30

This DExH helicase has received little attention until recently; early studies in mice revealed that the *Dhx30* gene is essential for survival, as complete *Dhx30* ko mice die early in embryonic development (Zheng et al., 2015). Several transcript variants arise due to the use of alternative promoters, and possibly also alternative splicing. Variations in the N-termini allow for either import into mitochondria or targeting to the cytosol of the expressed protein (Lessel et al., 2017; Bosco et al., 2021). As a consequence, a substantial portion of the protein resides in mitochondrial RNA granules which play a role in RNA processing and biogenesis of mitochondrial ribosomes (Antonicka and Shoubridge, 2015). Like several other DExH-type helicases, DHX30 carries additional domains besides the two RecA domains which constitute the helicase core. There are two dsRBDs in the N-terminal part of the protein, and the winged helix, ratchet like and OB fold domains in the C-terminus which are typical for DExH helicases.

We have previously established *de novo*, heterozygous, *DHX30* missense variants, affecting highly conserved residues within its HCMs, as a cause of a severe neurodevelopmental disorder, Neurodevelopmental disorder with variable motor and language impairment (NEDMIAL; #OMIM 617804) (Lessel et al., 2017). This condition is primarily characterized by severe global developmental delay (GDD), intellectual disability (ID), absent speech or speech limited to single words along with severe gait abnormalities (if walking is acquired at all). In contrast, individuals harboring a loss-of-function (frameshift or nonsense) variant develop a milder clinical course (Mannucci et al., 2021). The latter individuals have a mild GDD and ID, learn to speak full sentences and learn to walk in the second year of life. Two other missense variants outside HCMs have been described which are associated with a different clinical course. However, their causality still remains to be fully confirmed.

By performing *in-depth* functional analyses we were able to provide a molecular understanding for this genotype–phenotype correlation. Missense variants within the helicase core motifs (HCMs) of DHX30 impair either its ATPase activity or RNA binding capacity, and thereby its RNA helicase activity (Lessel et al., 2017; Mannucci et al., 2021). However, in addition to this clear loss-of-function, these missense variants additionally lead to a detrimental gain-of-function by inducing stress granule (SG) formation with concomitant global translation impairment. Utilizing CRISPR/Cas9 based technology, analyses of two DHX30 knockdown/knockout models, HEK293T cells and zebrafish model, revealed an impairment of SG formation (Mannucci et al., 2021). These data strongly suggest that the severe DHX30-associated phenotype (NEDMIAL) is due to the selective gain-of-function by triggering SG formation.

### DDX3X

The gene coding for the DEAD-box helicase DDX3X is localized on the X-chromosome and is one of the few genes known to escape X inactivation in females; a second gene on the Y-chromosome codes for the almost identical DDX3Y protein, apparently ensuring equal gene dosage for this type of helicase in males (Lahn and Page, 1997). Germline variants in *DDX3X* are one of the most common causes for intellectual disability in females; indeed more than a hundred affected individuals have been reported (Snijders Blok et al., 2015; Lennox et al., 2020; Greene et al., 2023). Variants in *DDX3X* are associated with a wide spectrum of neuronal phenotypes, ranging from ID and loss of speech to severe failures of cortical development including microcephaly and polymicrogyria (a condition characterized by too many, but too small folds in the surface of the cortex). In most cases, females carry *de novo*, heterozygous loss-of-function or missense variants. In addition, hemizygous variants inherited from unaffected mothers have also been identified in rare male patients. Missense variants lead to a significantly more severe outcome, suggesting a dominant effect of these variants (Lennox et al., 2020). Similar to the situation in *DHX30*, several missense variants alter residues in one of the conserved HCMs; thus, there are four cases with a severe phenotype carrying the T532M variant in motif VI. In addition, there is a number of cases with variants outside of the HCMs but within the two RecA helicase core domains. Functional analysis shows that lack of ATPase and RNA helicase activity in missense variants correlates with severity of disease (Lennox et al., 2020).

Studies of the mammalian DDX3X protein, as well as the yeast homolog Ded1p, suggest that this helicase is involved in translation and may play a role in unwinding complex 5′ untranslated regions (5’UTRs) during scanning of the preinitiation complex (Chuang et al., 1997; Hilliker et al., 2011). Work by Soto-Rifo et al. (2012) suggested that DDX3X needs to unwind secondary structures close to the 5′ end which occlude the 5’cap structure, thereby preventing access of the cap binding protein eIF4F. An additional role in the formation of stress granules was also discussed which might be directly related to the role of DDX3X in translation (Hilliker et al., 2011). Interestingly, somatic missense variants found in medulloblastoma, as well as germline variants in NDD patients, lead to excessive formation of stress granules even in the absence of cellular stressors such as heat or oxidative stress (Valentin-Vega et al., 2016; Lennox et al., 2020). In this respect, pathogenic variants in *DDX3X* to some extent mimic missense variants in *DHX30* which also lead to enhanced stress granule formation [see above (Lessel et al., 2017; Mannucci et al., 2021)]. However, a recent analysis in neuronal progenitor cells suggested that some of the NDD-associated missense variants trigger formation of ribonucleoprotein (RNP) granules that may not be stress granules (Lennox et al., 2020).

In the mammalian brain, lack of DDX3X leads to reduced neurogenesis during embryonic development, likely explaining the polymicrogyria (Lennox et al., 2020). A conditional knockout line in mice with deletion of Ddx3x expression in neural progenitor cells in early embryonic development showed that the encoded protein is necessary for cell cycle control and for the generation of a sufficient number of neuronal cells. DDX3x does so by promoting translation of a small set of mRNAs relevant for neurogenesis (Calviello et al., 2021; Hoye et al., 2022). Similar defects in cortical neurogenesis were observed in mice lacking another helicase gene, EIF43/DDX48, suggesting a common pathological mechanism (Lupan et al., 2023). In zebrafish, mutant *Ddx3x* causes a deficit in the Wnt signaling pathway (Snijders Blok et al., 2015). A previous study had shown that the DDX3X protein performs some “moonlighting” in this pathway as an essential positive regulator of casein kinase 1 (CK1ε), which is required for phosphorylation of Disheveled and activation of the transcriptional role of β-catenin (Cruciat et al., 2013). This raises the question, whether the helicase activity or the CK1ε-dependent activity of DDX3 is relevant for human pathologies. Activation of CK1ε by DDX3 does not require RNA binding or helicase activity of the DDX3 protein, as many of the conserved motifs involved in RNA or ATP binding can be deleted without affecting CK1ε binding or activation of the signaling pathway. Instead, the DDX3X function in this pathway depends on its ability to interact with CK1ε though a sequence element in its C-terminus (Cruciat et al., 2013). Thus, current evidence indicates that a disrupted RNA helicase function of DDX3X, primarily caused by pathogenic variants in the helicase core region, is the main cause of NDD in patients.

### DDX6

The *DDX6* gene (MIM: 600326) encodes the DEAD-box helicase 6, involved in the regulation of mRNA decay and translation. DDX6 is an essential component of P-bodies, cytoplasmic granules containing enzymes necessary for the post-transcriptional regulation of mRNA. In fact, DDX6 is one of the very few proteins which are actually essential for P-body formation (Weston and Sommerville, 2006; Ayache et al., 2015). An initial publication from 2019 reported five *de novo* missense variants in DDX6 in individuals with neurodevelopmental disorders (Balak et al., 2019). All the variants were located in the same exon of the gene and affected four amino acids from two conserved motifs (amino acids 372–373 and 390–391) of the second RecA domain of the protein, namely the QxxR domain and the motif V. These variants affect the ability of DDX6 to form P-bodies and to interact with several of its partners involved in translation control. All individuals present with global developmental delay, intellectual disability, hypotonia, gait instability with a delay in walk acquisition, and similar dysmorphic features including a high-bossing forehead, bulbous nasal tip, hypertelorism, epicanthus, arched eyebrows and low-set ears, associated with a small head circumference. They also present with additional non-neurological symptoms, such as cardiac, hand/foot, and urogenital anomalies. The identification of additional nonsynonymous variants in individuals with NDD will be necessary to refine the syndrome associated with *DDX6* variants and to establish if missense changes located outside the QxxR and the motif V domains and truncating variants might also be pathogenic. So far, little is known about the role of DDX6 in the brain and how its dysfunction could alter normal processes of brain development, but few studies have reported its involvement in neuronal differentiation and synaptic plasticity. In mouse neural stem cells, DDX6 is needed for neuronal differentiation by regulating let7a activity through cooperation with TRIM32 (Nicklas et al., 2015). DDX6 also regulates the retinoic acid-induced neuronal differentiation of human neuroblastoma cell lines SH-SY5Y and SK-N-SH (Shih et al., 2023). Finally, a role of DDX6 in mediating NMDAR-dependent spine shrinkage via the Ago2 dependent silencing of Limk1 has been recently described in rat neurons (Perooli et al., 2024).

### DHX9

DHX9 is involved in transcription, in the regulation of R-loops and in the repair of DNA double strand breaks by BRCA1 (Chakraborty et al., 2018; Cristini et al., 2018). In addition DHX9 represses the effects of Alu elements in the human genome on RNA processing (Aktas et al., 2017). For these purposes, DHX9 needs to be targeted to the nucleus by a nuclear localization sequence (NLS) in the C-terminal part of the protein. However, recent work in hippocampal neurons has shown that a substantial part of the protein may be present in the cytosol, specifically associated with the dendritically localized mRNA coding for Dendrin (Yang et al., 2024). Dhx9 deficient mice are viable but display distinct behavioral and neurological abnormalities (Calame et al., 2023). Although, OMIM still does not list *DHX9* as associated with human disease, two very recent studies identified pathogenic variants in *DHX9* in human patients. Thus, actually already formally establishing the link to NDD. Again, some variants associated with a moderate NDD phenotype alter conserved residues in motif I or motif VI and interfere with ATPase activity (Calame et al., 2023; Yamada et al., 2023). More severe NDD is observed with variants which alter the NLS and interfere with nuclear localization of DHX9; these lead to a higher number of R-loops and double strand breaks, indicating that the function of DHX9 in these processes is indeed required for neuronal homeostasis and function. Adding to phenotypic complexity, Calame et al. (2023) also identified missense variants in the winged helix and the C-terminal RGG domains of DHX9 which associate with hereditary motor and sensory neuropathy (also known as Charcot–Marie Tooth type 2 disease).

## Conclusion

The recent identification of pathogenic variants in several genes coding for DExH/DEAD-box RNA helicases has raised a strong interest in the function of this group of enzymes. Due to extensive studies in model organisms such as yeast, the functional relevance of the conserved helicase core domains has been elucidated in much detail (Linder and Jankowsky, 2011). These data from basic science strongly aided the interpretation of individual missense variants found in patients. Thus, it became clear that quite often the genetic variants affected key residues in HCMs, thereby interfering with the RNA binding, ATPase and eventually helicase activity of the encoded proteins. Nevertheless, in many cases it is still unclear whether a specific variant identified in a patient is pathogenic, or a harmless polymorphism. Inexpensive, non-complicated assays are needed to assess molecular relevance. Furthermore, there is still a large gap between understanding the molecular relevance of individual genetic variants, and understanding the relevance of these variants on a neuronal or systems level. Quite often, the particular cellular process which is relevant for disease is unknown. Thus, while DHX30 is partially present in mitochondria (Antonicka and Shoubridge, 2015; Bosco et al., 2021), the phenotypes of patients carrying variants in helicase core motifs of DHX30 are not typical for a mitochondrial disorder (Lessel et al., 2017). As the function of non-mitochondrial DHX30 is currently unknown, it is difficult to determine why variants in this gene lead to such a severe phenotype. In the case of DDX3X, most data now point to the relevance of translational control, as this helicase is needed for efficient translation of mRNAs with longer, structured 5’UTRs (Hoye et al., 2022). Nevertheless, it is unclear how the role of DDX3X in Wnt signaling may contribute to the development of intellectual disability in carriers of pathogenic variants. Further work both in mouse models as well as in induced pluripotent stem cell models derived from patient cells will be necessary to determine which particular aspects of neuronal RNA metabolism are affected by particular variants in DEAD-and DExH-box helicases.

Finally, it is mostly unclear how treatment can be achieved for these very rare disorders. Small molecule inhibitors have been identified for some helicases (reviewed by Naineni et al., 2023), which may be helpful in cases where the pathogenic variants cause a clear gain-of-function. Alternatively, specific antisense oligonucleotides may be considered in cases where a missense variant causes a more severe phenotype than the loss-of-function variants (as observed in DHX30; Mannucci et al., 2021).

## Author contributions

JL: Writing – original draft, Data curation, Formal analysis, Visualization. SD: Formal analysis, Visualization, Writing – original draft. AP: Writing – original draft, Conceptualization, Writing – review & editing. DL: Conceptualization, Writing – original draft, Writing – review & editing, Data curation, Funding acquisition, Supervision. H-JK: Conceptualization, Funding acquisition, Supervision, Writing – original draft, Writing – review & editing.

## Footnotes

^1^ www.omim.org

^2^ www.genenames.org

^3^ gnomad.broadinstitute.org

^4^ hbatlas.org

## Supplementary material

Supplementary Figure S1 Helicase core motifs of human DEAD box helicases.

Supplementary Figure S2 Helicase core motifs of human DExH helicases.
