# Supplementary figures and images for: The role of DEAD- and DExH-box RNA helicases in neurodevelopmental disorders
Source: Front Mol Neurosci. 2024 Aug 1;17:1414949. doi: 10.3389/fnmol.2024.1414949 (PMC11324592; doi:10.3389/fnmol.2024.1414949)

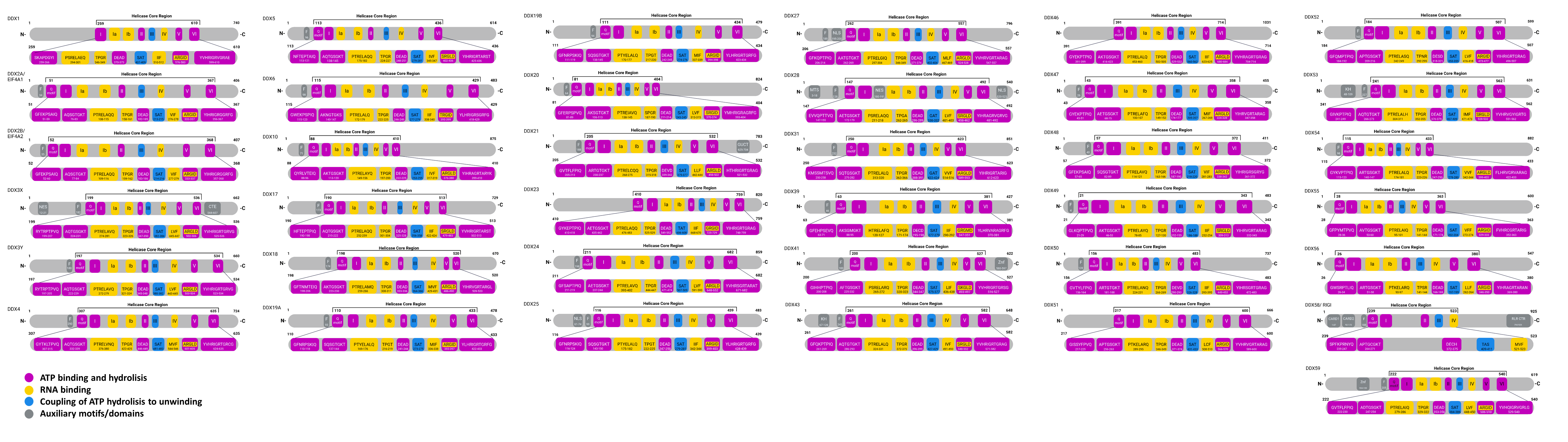

Supplement: Supplementary Figure S1 — Helicase core motifs of human DEAD box helicases. [file Image_1.tif]

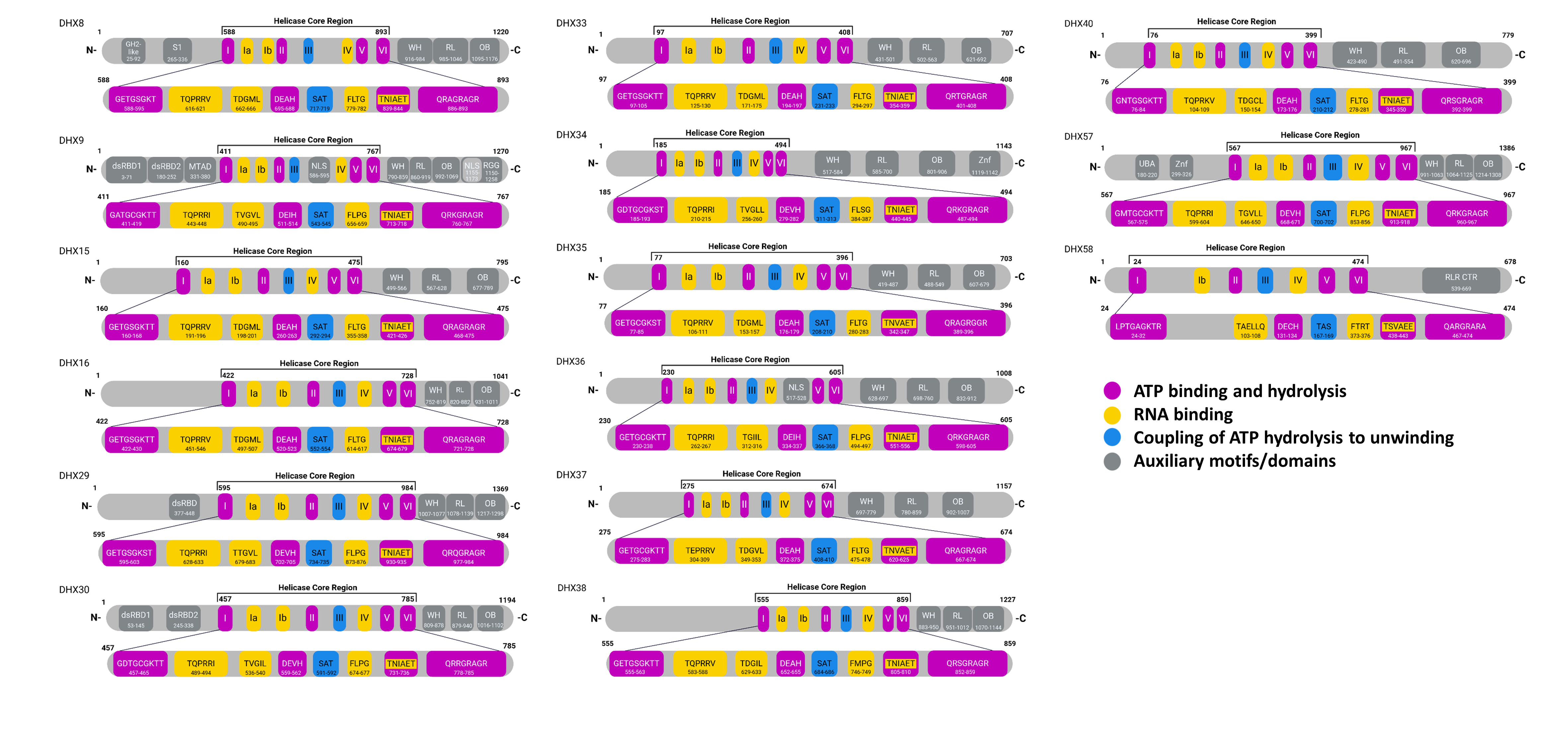

Supplement: Supplementary Figure S2 — Helicase core motifs of human DExH helicases. [file Image_2.tif]
